# Re-engineering a Clinical Trial Management System Using Blockchain Technology: System Design, Development, and Case Studies

**DOI:** 10.2196/36774

**Published:** 2022-06-27

**Authors:** Yan Zhuang, Luxia Zhang, Xiyuan Gao, Zon-Yin Shae, Jeffrey J P Tsai, Pengfei Li, Chi-Ren Shyu

**Affiliations:** 1 National Institute of Health Data Science Peking University Beijing China; 2 Advanced Institute of Information Technology Peking University Hangzhou China; 3 Department of Statistics University of Missouri Columbia, MO United States; 4 Department of Computer Science and Information Engineering Asia University Taichung Taiwan; 5 Institute for Data Science and Informatics University of Missouri Columbia, MO United States

**Keywords:** blockchain, clinical trials, clinical trial management system, electronic data capture, smart contract

## Abstract

**Background:**

A clinical trial management system (CTMS) is a suite of specialized productivity tools that manage clinical trial processes from study planning to closeout. Using CTMSs has shown remarkable benefits in delivering efficient, auditable, and visualizable clinical trials. However, the current CTMS market is fragmented, and most CTMSs fail to meet expectations because of their inability to support key functions, such as inconsistencies in data captured across multiple sites. Blockchain technology, an emerging distributed ledger technology, is considered to potentially provide a holistic solution to current CTMS challenges by using its unique features, such as transparency, traceability, immutability, and security.

**Objective:**

This study aimed to re-engineer the traditional CTMS by leveraging the unique properties of blockchain technology to create a secure, auditable, efficient, and generalizable CTMS.

**Methods:**

A comprehensive, blockchain-based CTMS that spans all stages of clinical trials, including a sharable trial master file system; a fast recruitment and simplified enrollment system; a timely, secure, and consistent electronic data capture system; a reproducible data analytics system; and an efficient, traceable payment and reimbursement system, was designed and implemented using the Quorum blockchain. Compared with traditional blockchain technologies, such as Ethereum, Quorum blockchain offers higher transaction throughput and lowers transaction latency. Case studies on each application of the CTMS were conducted to assess the feasibility, scalability, stability, and efficiency of the proposed blockchain-based CTMS.

**Results:**

A total of 21.6 million electronic data capture transactions were generated and successfully processed through blockchain, with an average of 335.4 transactions per second. Of the 6000 patients, 1145 were matched in 1.39 seconds using 10 recruitment criteria with an automated matching mechanism implemented by the smart contract. Key features, such as immutability, traceability, and stability, were also tested and empirically proven through case studies.

**Conclusions:**

This study proposed a comprehensive blockchain-based CTMS that covers all stages of the clinical trial process. Compared with our previous research, the proposed system showed an overall better performance. Our system design, implementation, and case studies demonstrated the potential of blockchain technology as a potential solution to CTMS challenges and its ability to perform more health care tasks.

## Introduction

Clinical trials are considered to be the cornerstone of the development of new drugs or treatments because they have investigated the safety and efficacy of new therapeutics using a standard protocol [1]. As conducting clinical trials involves complex processes, good management is critical for success [2]. The clinical trial management system (CTMS) is a set of software tools used for managing clinical trial processes including but not limited to protocol development, site selections, patient recruitment, study conduct, data collection, data analysis, and study closeout. With the increasing adoption of the CTMS, many substantial benefits such as accessing up-to-date information, improving data quality, and boosting overall study efficiency have simplified the traditional labor-intensive management process [3-5]. A complete CTMS design must be secure, cost-efficient, compliant with regulations, traceable, and auditable to manage the process in each phase of the study [5-9]. However, the current CTMS market is fragmented and lacks thorough designs with all the required features and management tools [2,7]. According to the 2019 Unified Clinical Operations Survey provided by Veeva (a global life science service), nearly all respondents (99%) had issues with their current CTMS, and 90% of the respondents reported a significant deficiency in at least 1 CTMS application [10,11]. Emerging technologies, such as blockchain, are believed to potentially re-engineer CTMSs and provide a comprehensive solution [12].

Blockchain is an open-source distributed ledger technology that has been proven in the areas of security, stability, and robustness in real-world applications, including cryptocurrencies [13-15]. A blockchain consists of continuously generated blocks containing validated transactions, time stamps, and block IDs used for chaining to the previous block. It is considered to be a revolutionary technology, as it has unique features such as immutability to ensure data consistency; a peer-to-peer system with public auditability (all blockchain transactions can be audited by any user at any time) to provide regulatory compliance; anonymity (all users are represented by a unique hash string) to protect patient privacy [16]; and a smart contract, which is a self-executing programmable computer protocol that can be designed for different applications. These features are a perfect fit for health care applications [17-19]. However, most blockchain designs used for health care applications remain in the conceptual stage, and there are several technical challenges such as scalability constraints [20-24]. Quorum blockchain, a private blockchain developed by JP Morgan that requires participating users to gain permissions from the blockchain initiator before joining, has enhanced security, scalability, and efficiency based on the original blockchain [25,26]. The performance of the Quorum blockchain in areas such as transaction throughput and transaction latency has been evaluated as extraordinarily improved (compared with the original blockchain) using the Raft consensus mechanism for the validation process without compromising its unique properties [25].

We have implemented a blockchain platform that provides unique software designs for key components of CTMSs to achieve better management and monitoring of clinical trials with the following applications: (1) an auditable, sharable, and transparent electronic trial master file (eTMF); (2) a fast patient recruitment model with an automated matching mechanism through the smart contract and a simplified enrollment using a digital signature validated by the blockchain; (3) a timely electronic data capture (EDC) system that ensures data consistency, traceability, and security through blockchain’s properties; (4) a reproducible data analytics module that keeps records of data and code use; and (5) a secure, auditable, and efficient payment and reimbursement model. We conducted case studies for each application to empirically prove its feasibility and test its scalability, stability, and efficiency.

## Methods

### Overview

**Figure 1:**
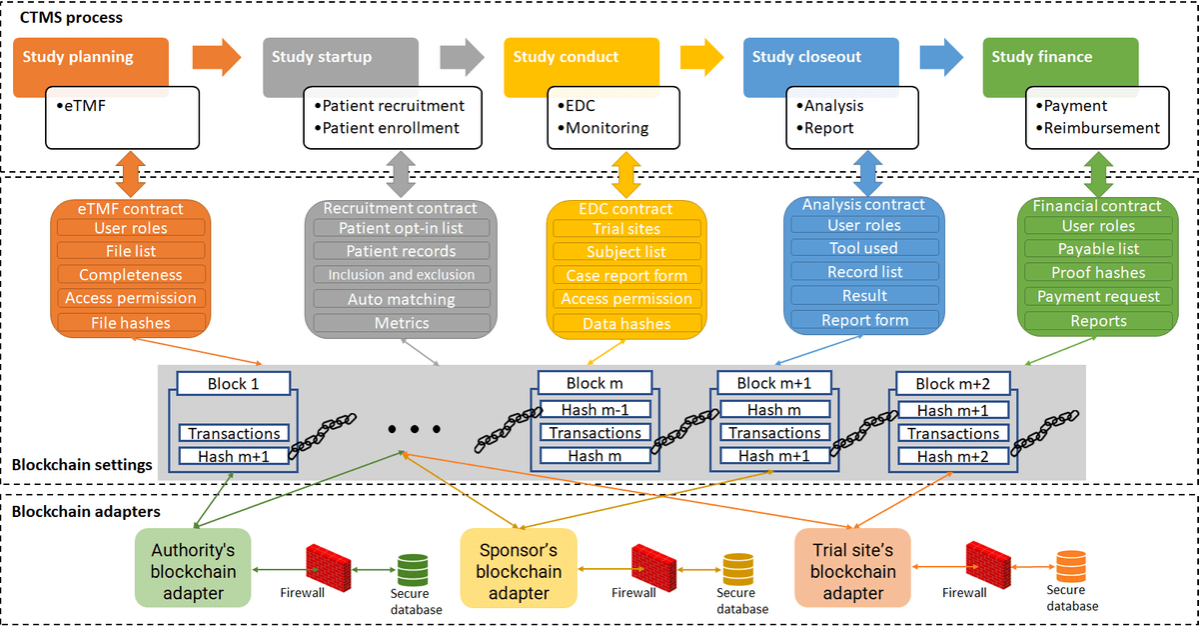
The overall architecture of 5 different clinical trial processes. Different applications are implemented by smart contracts defined through blockchain initiation. Participating sites require blockchain adapters to interact with the blockchain system and the secure database protected by local health information technology regulations. CTMS: clinical trial management system; EDC: electronic data capture; eTMF: electronic trial master file.

Figure 1 depicts the overall architecture and main smart contract designs that span five different stages of the clinical trial process: (1) study planning targets on the eTMF for clinical trial protocol development; (2) following the protocol’s establishment, study start-up focuses on recruiting participants for clinical trials; (3) while the clinical trial is in progress, study conduct develops EDC for data collection and monitoring the safety and efficacy of the treatment; (4) during the closing phase, study closeout collaborates with statistical tools to provide a reproducible analytics report; and (5) study finance adopts the blockchain’s nature of cryptocurrency for payment and reimbursement.

This architecture is generalizable to all different clinical trials; therefore, the participating site can use the same CTMS to manage simultaneous clinical trials by switching trial IDs obtained by the sponsors, whereas the registration on the blockchain-based CTMS remains constant. It is noteworthy to mention that CTMSs may require additional functions such as protocol development, which are not included in our system design, as the current procedures for protocol development are sophisticated enough [27] with no need to adopt a new approach such as blockchain to reinstate the existing process, although most present tools can be integrated with our proposed blockchain-based CTMS without extensive arrangement.

**Figure 2:**
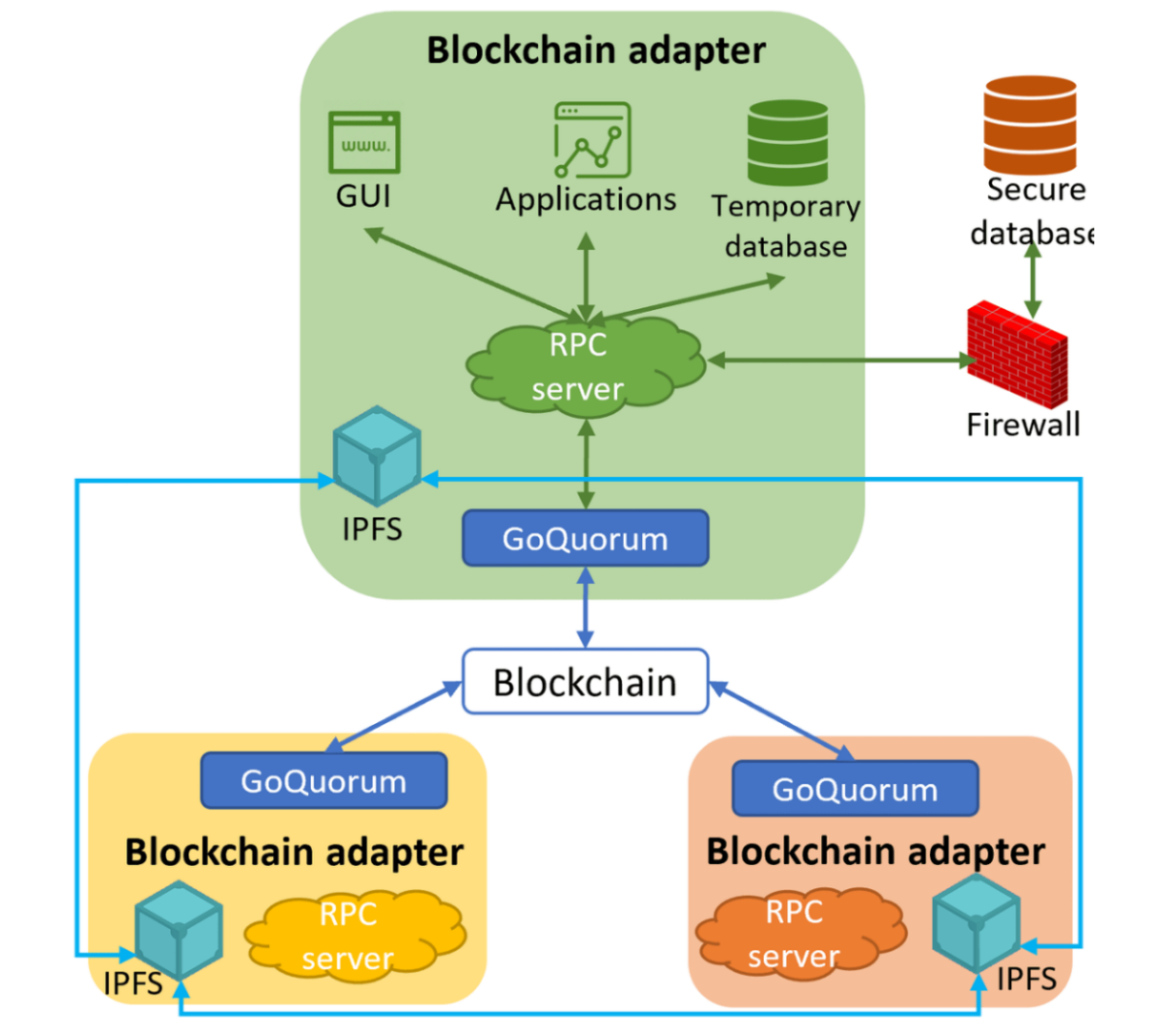
Blockchain adapter design and connections. All adapters have the same setup with an RPC server connecting local applications and databases, an IPFS that connects to other IPFS on each adapter, and a GoQuorum application programming interface that connects to the blockchain. GUI: graphical user interface; IPFS: interplanetary file system; RPC: remote procedure call.

The remainder of this paper is organized as follows: (1) the environment setup specifies the details of the hardware and software required to construct the system, notably the blockchain adapters shown in Figure 1; (2) the following sections describe the blockchain settings as smart contracts for each stage of the CTMS process, as shown in Figure 1; and (3) as shown in the *Results* section, we conducted case studies on study planning, study start-up, and study conduct to test the blockchain features and overall performance, such as scalability.

### Environment Setup

In this study, we used a laptop equipped with 16 GB of RAM, an i5 processor, and a 1 TB hard drive to represent the authority’s node and 5 Intel NUC machines, each equipped with 16 GB of RAM, an Intel i3 processor, and a 1.5 TB hard drive to represent the clinical trial sites’ and sponsors’ nodes. These machines were set up at 2 different locations under different networks. Owing to the regulatory compliance required by each participating site, we converted each blockchain node into a blockchain adapter that abides by local health information technology regulations [28]. As shown in Figure 2, each blockchain adapter installed the Ubuntu operating system, which in turn runs GoQuorum, an Ethereum-based Quorum blockchain client. Once the authority node started the client, the Quorum blockchain with the Raft consensus mechanism was built automatically. Then, the blockchain adapter will be added to the blockchain by the authority and will be able to communicate with other blockchain adapters as well as the local secured database protected by health IT when the participating site obtains permission to join the system. Tools can be installed on the blockchain adapter and integrated with the blockchain through a remote procedure call server. For example, a team of professionals such as medical experts, statisticians, clinical research coordinators, and medical writers can use the blockchain adapters for protocol development. Existing tools can still be used as anticipated. The sole exception (limited to development scenarios) was the ability to store a log file in the blockchain after each use. In all other aspects, users can take advantage of blockchain's unique features such as immutability to ensure file consistency and traceability to acknowledge the users who edited the file, as well as decentralization to improve the efficiency of working distributively without changing the existing legacy process. Each adapter has an interplanetary file system (IPFS) installed, which is an innovative peer-to-peer distributed file system. Each file stored in the IPFS is assigned a unique cryptographic hash for indexing and ensuring consistency. Compared with other distributed file systems, the IPFS has shown great improvement in efficiency, scalability, and stability [29]. However, the design concept of the IPFS lacks the capability of access control and file use tracking [30]. However, this makes it a perfect match for blockchain. The IPFS can be used for data storage, whereas blockchain serves as a content management system.

A unique public-private key pair will be generated for each user such as participants, investigator, sponsor, and others, after the user registers a blockchain account through a site’s blockchain adapter. Patients and potential participants must register on-site so that the administrators from the trial sites can prove their identities and map their local patient ID to the blockchain account with their consent. A hash value of the public key, also known as the blockchain account address, will be used to represent the user’s identity. A private key will be used as a digital signature. All transactions must be signed by the sender’s private key before they can be recorded in the blockchain. Each group, such as the financial management team, has an umbrella account in addition to separate individual user accounts, each of which maps to the umbrella account for each member so that the entire group can share permission when authentication to the group is made. Potential participants must visit trial sites to opt in to the system and generate their blockchain account so that the trial site can verify their identities. Instead of memorizing the key pair, a username and password or biometric authentication mechanism can be used on a graphical user interface (GUI) for users to log into the blockchain system.

To build the blockchain-based CTMS, we made the following assumptions: (1) each participating site, including the sponsor, trial sites, site institutional review boards, and the Food and Drug Administration, is required to provide at least 1 blockchain node, which can be any electronic device that can install the Quorum blockchain; (2) the authority (eg, Food and Drug Administration) has initiated the blockchain system so that all that the participating site requires is to obtain permission from the authority before joining the system by proving their identity; and (3) each participating site has an administrator to operate the system.

### Study Planning

**Figure 3:**
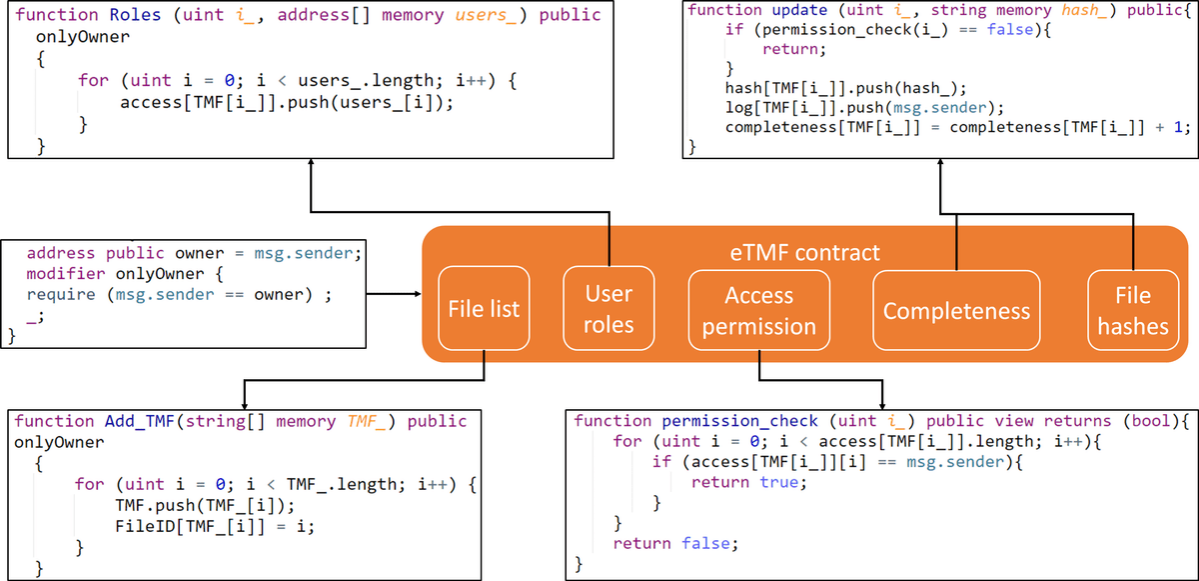
A portion of the source code of the electronic trial master file (eTMF) contract design. These codes show the main logic of each function. All smart contract functions are predefined, and users can use graphical user interfaces to call the functions.

With the increasing adoption of electronic documents for clinical trials, planning, sharing, and managing documents have become increasingly critical and intricate [31]. The eTMF is a form of content management system used to manage and collaborate in a timely fashion on essential clinical documents throughout the life cycle of clinical trials. However, several persistent challenges exist in most eTMF designs, such as the inability to audit unlocatable files; inaccurate metrics for timeliness, quality, or completeness; inconsistency caused by loss or alteration of the information; and collaboration issues caused by different trial master file (TMF) standards. Our eTMF design contains a smart contract used to control file access, validate file consistency, and manage collaboration in TMF development and the IPFS network used for file storage and file indexing. Figure 3 shows a portion of the source code of the smart contract for each function.

The TMF document list and other expected artifacts list must be identified in the eTMF smart contract at the beginning of the study planning phase. Sponsors must assign files to team members so that they can work jointly by adding their blockchain accounts to the smart contract associated with the file ID from each TMF. All TMFs are encrypted using OpenSSL and a randomly generated key pair before being stored in the IPFS [32]. All users can download the file from the IPFS using the file hash, but only users who have permission from the sponsor can retrieve the decrypt key from the smart contract to decrypt the file. When a team member works on a certain file, the blockchain adapter from the member’s site automatically sends a flag to the smart contract to block other team members from working on the same file. When the team member finishes editing the file, the blockchain adapter will encrypt the new version of the file with a random new pair of keys, upload the encrypted file to the IPFS, obtain a new hash value from the IPFS, and send the decrypt key, hash value, and negative flag to the blockchain to update the file registration information. The completeness metric (the percentage of expected artifacts that are completed) will be updated automatically.

Using blockchain technology for eTMF can provide the following unique features: (1) consistency—each version of a file will have a hash value stored in the blockchain, and any changes to the file will result in a mismatch of its new hash with the original hash; (2) traceability and auditability—each team member must work on the file sequentially so that any changes can be traceable to the editing user through blockchain transaction history [33] (users can audit who has changed the file by checking the log files in the blockchain, but only the sponsors, or the authority, know the real identity of the user); (3) efficiency—using IPFS as file storage is efficient compared with other file transferring processes because team members can collaboratively work on the same file; and (4) security—with blockchain’s security setting, all transactions are considered secure so that only the recipients can receive the correct decrypt key for the file.

### Study Start-up

**Figure 4:**
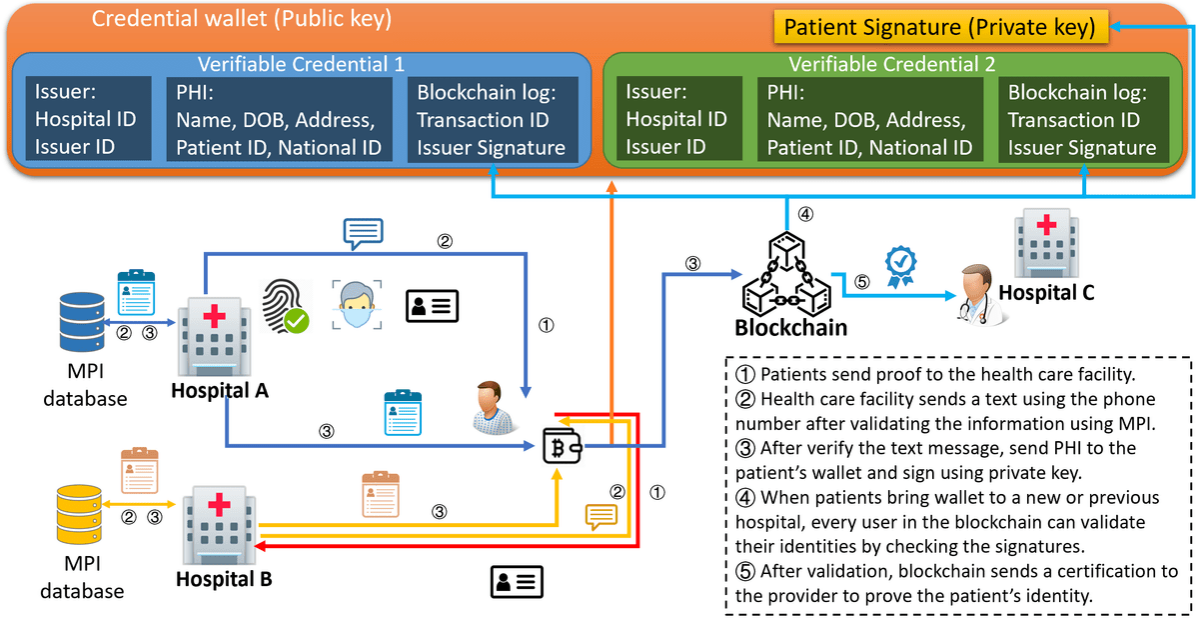
Trial sites must register participants and input primary medical history to the smart contract. The smart contract will automatically send notifications to the matched patients, asking for authentication through their mobile device using their fingerprint. DOB: date of birth; MPI: master patient index; PHI: protected health information.

After the study team has selected trial sites and defined target enrollment metrics, clinical trials must meet the recruitment goal. Patient recruitment has been recognized as a key to success. However, 86% of clinical trials fail to meet their recruitment goals on time. We refined our earlier work, which was a blockchain-based recruitment model using a smart contract for automated matching [13] for use under the CTMS study start-up scheme, as shown in Figure 4. We have developed a patient credential wallet for patients to store their key pairs as well as the credentials issued by the health care facilities on their local device. Verifiable credentials contain issuers’ information such as hospital ID and issuer ID, patients’ protected health information, and the ID of the blockchain transaction that was made and signed by the issuer when the patient presents their blockchain account on-site. In this configuration, each health care facility has the protected health information mapped to the patients’ public key, but the patients’ private keys are maintained locally.

Users who want to participate in clinical trials must follow the same procedure outlined for patients and participants. They also need to provide permission for the use of their electronic health records (EHRs) for future matching purposes. The hospital administrator must input the basic user information into the recruitment contract, including demographic information and primary diagnoses from past visits. As soon as the sponsor inputs the recruitment inclusion and exclusion criteria into the smart contract, the smart contract can automatically screen potential participants by matching the basic information. After the initial screening is accomplished, hospitals can perform precise matching by checking the full EHRs of matched users. When a user is fully matched, the sponsor will send a transaction to the user to ask for enrollment. Future on-site visits are still required, but the enrollment process can be operated by sending out the consent form and asking the user to sign it using their private key [34], which will send a confirmation transaction to the smart contract. The smart contract also contains personalized metrics such as time consumption, cost, and retention, used to evaluate the performance of the team in the recruitment process and the timeliness of decisions to increase productivity.

The features of blockchain technology are a great fit for the needs of recruitment and enrollment for the following reasons: (1) transparency can improve the awareness of clinical trials for patients, (2) auditability ensures the legitimacy of clinical trials, (3) anonymity protects patient privacy, (4) asymmetric encryption eases the process for patient enrollment, and (5) the automated matching mechanism operating via a smart contract can significantly reduce the time required for recruitment.

### Study Conduct

Data collection is one of the most important processes for the evaluation and monitoring of aspects of the experimental condition (eg, drug effect) as clinical trials are conducted. Compared with the traditional paper-based case report form (CRF), which serves the sole purpose of recording information, EDC systems are used to collect data electronically, reduce data errors, improve the efficiency of the collation process, and enable faster data access. However, there are several challenges faced by both the paper form and the EDC system, such as security concerns, data inconsistency, and untimely (slow) data input. All clinical trials were monitored, which was a process of data and safety monitoring. The Data and Safety Monitoring Board comprises a group of professionals from different fields such as biostatistics, medicine, and ethics, who monitor patient safety and treatment efficacy. The legacy data monitoring method is source data verification (SDV), which is resource intensive and accounts for up to 30% of the total clinical trial budget. We designed an EDC contract to effectively collect data, reduce the need for SDV, and monitor patient safety persistently.

**Figure 5:**
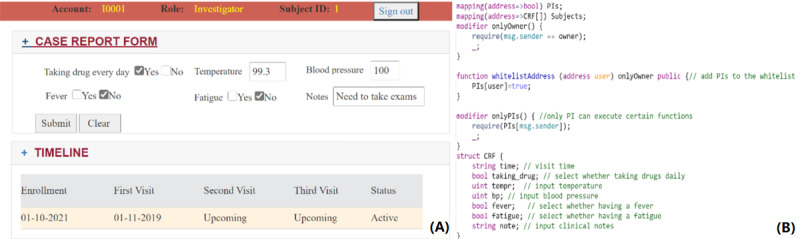
(A) The graphical user interface for principal investigators containing a sample electronic case report form (eCRF) coded through the smart contract and a sample timeline for the participant. (B) The smart contract is used for defining data fields and types of the eCRF. Blockchain adapters will retrieve the information from the smart contract and generate the eCRF.

**Figure 6:**
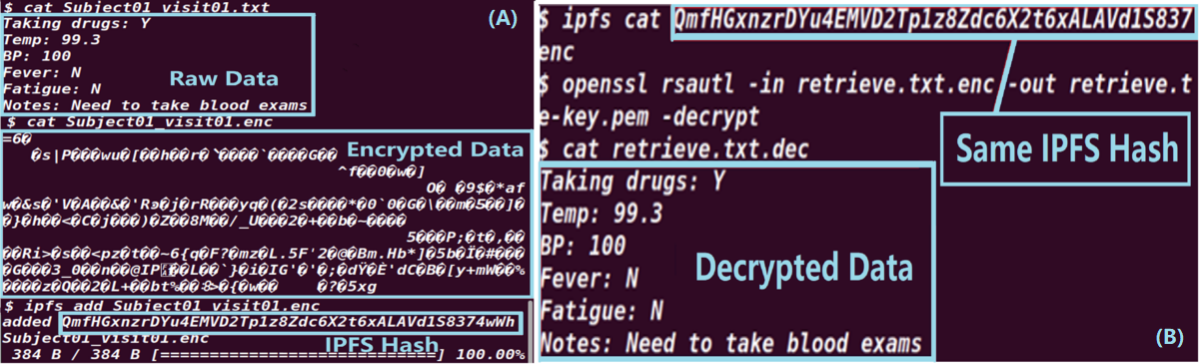
(A) The investigator’s blockchain adapter retrieves the data through the graphical user interface, encrypts the data using the investigator’s public key, and stores the encrypted data into the interplanetary file system (IPFS). (B) The sponsor’s blockchain adapter retrieves the encrypted data through the IPFS and decrypts the data using the private key.

After participants submit their consent to the blockchain during the recruitment phase, the system administrator from each trial site must register them in the participant list in the EDC contract to map their blockchain account to the trial ID and their local patient IDs. Figure 5A shows a customized CRF converted through a smart contract shown in Figure 5B. Data fields and types, such as selection and input, can be defined in the smart contract and retrieved by blockchain adapters for conversion into a GUI-based CRF. After each participant’s site visit, the investigator needs to input the records into the electronic CRF (eCRF). The records will then be automatically encrypted, hashed, and stored in the IPFS using the blockchain adapter of the site. Figure 6 shows the encryption, storing, and retrieving process after the data are input through the GUI. The smart contract will validate whether the trial site has permission to store the participant’s data, after which the visit ID and decrypt key will be sent through Quorum blockchain’s private transaction. This ensures that the data contained in the private transaction are encrypted, and only the recipient can decrypt using their private key, or the information can be made available to the sponsor by the site’s administrator. The sponsor’s blockchain adapter will automatically retrieve the decrypt key and hash from the blockchain, decrypt the records, and hash the records to compare with the hash stored in the blockchain. Mismatching hashes will create an alert to the trial site and for the sponsor to initiate further investigation. This can eliminate data inconsistency caused by falsification. However, most EDCs require manual input, and human data errors can also cause a data inconsistency issue. We have implemented a data extraction application on each blockchain adapter to automatically extract the CRF-required data from the visit records before storing it in the secured EHR database to reduce the risk of human errors. However, most CRFs have partial data fields that are trial oriented and are not included in the EHR, meaning that manual input is still needed. Although blockchain’s immutability features were intended to be designed as unchangeable for all records, some modifications may still occur owing to unintentional human error. However, the updated (erroneous) records cannot replace the previous input and will contain a pointer to the former hash of the data record for future validation. In this blockchain-based CTMS system, safety monitoring relies on the investigators to report through the EDC so that the safety monitoring team can evaluate only true issues of data and safety.

In this module, using blockchain and an IPFS for EDC has the following benefits: (1) immutability ensures data consistency from the data input through data analysis to reduce the need for SDV; (2) traceability improves the auditability as to who, when, and how the records were changed; (3) the efficiency of the IPFS permits fast data retrieval; and (4) the security property of blockchain protects patient privacy and data security. With the addition of the automated extraction mechanism added to the blockchain adapters, the efficiency and accuracy of the data collection process are significantly enhanced.

### Study Closeout

When the last participant completes their site visit, the clinical trial will enter the closeout phase. There will be a closeout checklist that can be collaboratively completed by sponsors and the team using the eTMF. The clinical trial database can be locked to prevent future changes after validation of the final data. Statistical analysis must be conducted to evaluate the outcomes of clinical trials. In the blockchain-based CTMS system, we created several R scripts for several statistical models in each blockchain adapter and added the names of the available statistical methods in the smart contract. The statisticians can use the existing script or use their preferred statistical tool to analyze the final data, after which they can generate the final statistical report. The source code must be encrypted and stored in the IPFS for validation purposes. The team members or authority can request the decrypt key from the sponsor and reproduce the results using the source code and clinical trial data.

Barriers to analyzing clinical trials are mainly those of selective reporting [35], incomplete reporting data [36], and a lack of appropriate statistical methods [37]. Blockchain provides solutions to the challenges in this stage through its immutability and auditability features, which help to ensure the completeness of reports. The analyzed data and applied methods will store a log file in the blockchain so that the study group and the authority can reproduce the results at any time to validate the completeness and appropriateness of audits of the analyzing methods.

### Study Finance

Numerous components can add to the cost of a clinical trial, such as regulatory services, start-up, and medical writing, of which all can create challenges in financial management. In this module, we use payment and reimbursement to the trial sites and patients [38] as an example of the potential use of blockchain technology as a financial management tool. The validation of the payment or reimbursement requests as to when and how the recipient is paid is a time-consuming process, making on-time payments challenging [39]. In this module, we designed a smart contract and a collaborative validation network in the blockchain-based CTMS.

Before a clinical trial begins, the study team should define a list of payable entities (as well as payable items) and input this list into the smart contract. This can standardize the payable items and reduce the risk of hidden fees. Each trial site may have different rates for the same payable item. The rates must also be defined through a smart contract accessible only by the sponsor and trial site. Compensation for the patient is normally based on the time required for the participant to take part in the study. After each visit, the trial site must send a request transaction containing the time spent and the payable items to the blockchain, store the encrypted proof in the IPFS, and send the decrypt key and hash to the sponsor. The clinical trial financial management team can validate the proof and send the payment requests to the sponsor. A transaction that contains a payment receipt will be sent from the sponsor to the trial site and marks the status of the request as paid in the trial site’s GUI. The payment to or reimbursement of the trial site has a similar process, as trial sites send request transactions that contain payable items to the sponsor and wait for the approval. However, payable items may not cover all requested payments. Trial sites need to follow the same request process with *additional items* in the payable items. Sponsors can collaborate to validate the proof and price the additional items to make payments.

Using blockchain technology for financial management has the following benefits: (1) a customizable charging standard for different trial sites as long as the sponsor agrees (all payable items and rates are preferred to be defined in the smart contract for an expedited validation process); (2) the traceability feature ensures that all requests and payments are traceable by the requester and the recipient (all the proof needs to be stored in the IPFS); (3) the immutability feature ensures that the request, payment, and proof of payment are not modifiable after the payment is made; and (4) the security property of blockchain protects user privacy.

## Results

### Overview

We implemented the blockchain-based CTMS and installed it on 6 blockchain nodes representing 1 authority, 2 sponsors, and 3 trial sites. Each blockchain node has been converted into a blockchain adapter. We generated 2 clinical trials with 1000 participants at each trial site for each study. We conducted 3 case studies to simulate the processes described in the study planning, start-up, and conduct sections to evaluate the feasibility and performance of the system. These studies were also conducted to assess the key components of the processes discussed in the *Study Closeout* and the *Study Finance* sections, such as ensuring the consistency of data recorded from statistical tools and reimbursement or payment forms.

### Study Planning

During this stage, the key benefit of the blockchain system is to record all changes in the essential files and ensure file consistency. The case study simulates the TMF collaboration process, as all experts are working on the same file named *protocol.txt* and sharing an *umbrella ID* for encryption purposes. The goal of this case study is to test the capability of (1) handling file conflicts while collaborating on master files, (2) ensuring traceability and auditability of file changes using blockchain’s properties, and (3) storing files into and retrieving files from the IPFS efficiently. We created 2 accounts for each node representing the 2 experts from each participating site to simulate the TMF collaboration process. The script is designed as follows: (1) an expert retrieves the file hash from the smart contract and the file from the IPFS using the hash, (2) the expert writes their blockchain ID (for tracing validation purposes) to the file and keeps the file open for 10 seconds, (3) encrypts the file and stores the new hash to the smart contract, and (4) repeats this script 20 times. The script is deployed on each blockchain adapter, and all scripts run simultaneously. If the file is being opened, the file will not be retrievable. In this case, the script will keep running until successfully executed.

**Figure 7:**
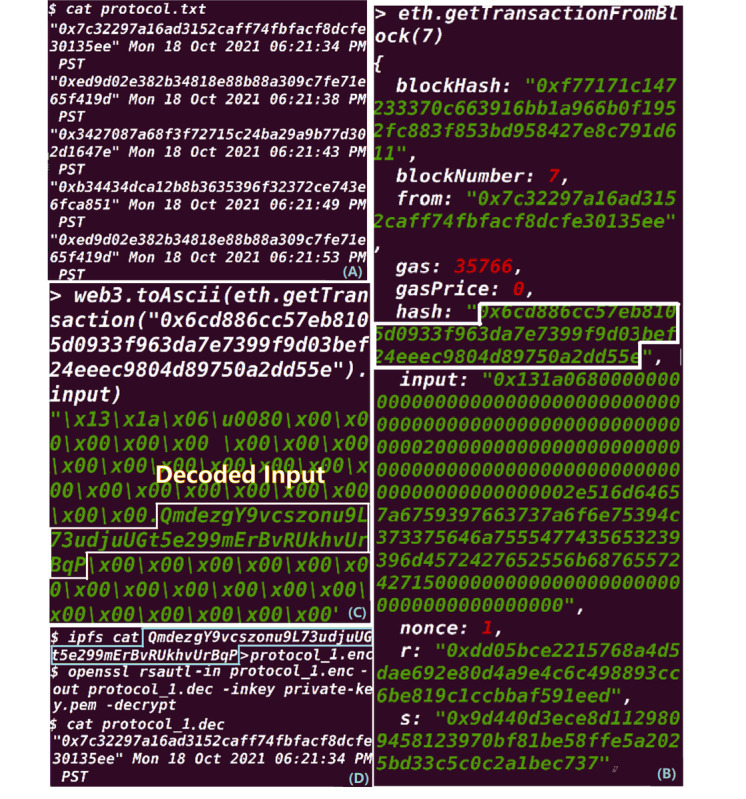
(A) Part of the final master file with all experts’ blockchain IDs and input times. (B) Trace the input record from the blockchain by checking the block number and transaction ID. (C) Decode the details of the transaction input. (D) Retrieve and decrypt the file using the hash stored in the blockchain transaction.

After 12 minutes and 38 seconds, the scripts were successfully executed, and all 60 records were moved into the final protocol file as shown in Figure 7A. Records can be traced from the blockchain by tracing transactions. For example, the first record is recorded in the transaction inside block 7 as shown in Figure 7B, where we can extract the transaction ID from the block and check the details using the ID, as shown in Figure 7C. Then, the encrypted file can be retrieved from the IPFS using the hash stored in the transaction and decrypted using the decrypt key under the umbrella ID as shown in Figure 7D.

### Study Start-up

In addition to blockchain features such as transparency, auditability, and anonymity, the key contribution of this module is to provide an automated matching mechanism that can filter out the potential participants that matched the recruitment criteria. We have repeated the case study from our previous recruitment work [13] using the Quorum-based approach to assess the accuracy and performance and comparing it with our previous Ethereum-based approach. The case study simulates the participant matching and recruitment process. The goal of this case study is to test the accuracy and efficiency of an automated matching engine and anonymity during the recruitment process.

We selected 6000 patient records of breast cancer from the Surveillance, Epidemiology, and End Results database and evenly distributed them into 3 clinical sites. Common inclusion and exclusion criteria from 10 recruiting clinical trials for breast cancer were selected to simulate the recruitment process. We created a script to populate the smart contract with criteria and patient records, such as demographic information and primary diagnosis. After calling the automated matching function, a total of 1145 of 6000 patients’ blockchain accounts were matched in 1.39 seconds, which was slightly better than the 2.13 seconds that resulted from our previous Ethereum approach.

### Study Conduct

The case study simulated the data collection process during a clinical trial using a sample eCRF designed through a smart contract. A script was created to mimic the data capture process: (1) the script randomly generated data for the data fields defined by the eCRF from the 3 trial site adapters; (2) the trial site adapters encrypt the data file using a random public key, store the encrypted data file in the IPFS and obtain the hash value, and send decrypt key and hash value to the sponsor through a private transaction; and (3) the sponsors’ adapters retrieve data from the IPFS and decrypt the data files. The goal of this case study is to test the following aspects: (1) data consistency from the input to the retrieval, (2) the successful rate and accuracy of the transactions for data collection, and (3) the scalability and efficiency of the system.

**Figure 8:**
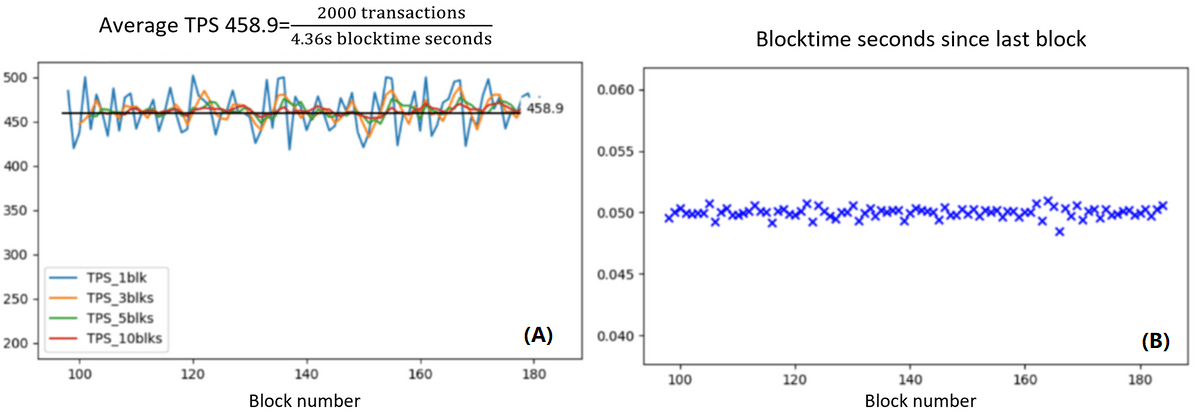
Scalability and stability test results of the first 2000 simultaneous transactions. (A) Transactions per second (TPS) values were calculated using every 1, 3, 5, and 10 blocks. (B) Time consumption of generating a new block.

We ran the script on each participant from each blockchain adapter every second for an hour. There were 1.2 million transactions written into the blockchain with an average latency of 1.73 seconds and 335.4 transactions per second (TPS), a key measurement of blockchain scalability. The remainder of the transactions were held in the buffer to sequentially push them into the blockchain. It took nearly 18 hours to send 21.6 million transactions generated by the script into the blockchain with a 100% success rate. All records have been precisely collected. Figure 8 shows the blockchain performance after submitting 2000 transactions simultaneously. The average TPS was 458.9 (SD 21.224), but it gradually decreased and stabilized during the simulation. The TPS was not associated with the block generation time from our simulation results.

As script 3 is purely off-chain, the stability is based on the performance of the IPFS and the specifications of the adapter’s devices. We did not included script 3 in our stability test, as many researchers have proven the performance of the IPFS [40]. To test system robustness, we manually shut down the sender’s blockchain adapter after the transaction and found that the recipient could still retrieve the data.

## Discussion

### Principal Findings

In addition to the scalability test, we have evaluated various blockchain features that are critical at different stages of a clinical trial. Blockchain demonstrates auditability, transparency, and immutability in the *Study Planning* section. We manually submitted malicious transactions to tamper with the current eTMF using a random blockchain account outside the umbrella ID. These transactions were automatically filtered out by the blockchain with no responses. All transactions are publicly auditable, and the recorded data cannot be changed. From our simulation, blockchain plus IPFS is also efficient for file storage and retrieval. In the study start-up case study, we mainly tested the feasibility and efficiency of subject matching through smart contracts. The simulation results show that the smart contract can match potential participants accurately and efficiently without exposing the patients’ identities. During study conduct case study, we evaluated data consistency and scalability efficiency and robustness of the blockchain. TPS is a key measurement of blockchain’s scalability, and Quorum blockchain shows a better performance compared with Ethereum. All legitimate transactions have been successfully executed and recorded in the blockchain. The blockchain also shows robustness when a single node fails in our simulation.

### Limitations

The main limitation of the proposed architecture is that health care facilities must cooperate to provide blockchain adapters to join the system. As blockchain adapters need to communicate with secure databases protected by local health care facilities’ firewalls and store classified documents and patient records outside the firewall to the IPFS, health care facilities need to follow the local health information technology regulations to set up the blockchain adapters. Although there are no hardware requirements for blockchain adapters, the device specifications may affect their performance. From our simulation experience, too much transaction generation may take up memory and crush the blockchain node. From a previous study that evaluated the scalability of Quorum blockchain using powerful cloud service as 8 blockchain nodes, their testing result of 8 nodes with Raft consensus mechanism has a similar TPS with slightly lower latency which is 1.4 seconds compared with our 1.7 seconds [41]. Both studies have empirically proved the Quorum blockchain as stable, robust, and scalable. Another limitation is that the designed eCRF is intended only for simulation purposes, and the data are randomly generated to test the scalability of the system. The real eCRF may have more complex designs, but because data are collected and transferred through the IPFS while blockchain only serves as a key distributor, access controller, and log auditor, there should not be significant changes in the sizes of blockchain transactions that cause concerns about the feasibility, scalability, and stability of the blockchain system.

### Future Work

Our future work will continue to investigate the needs of the clinical trial process and add more comprehensive functions to the proposed blockchain-based CTMS architecture, such as adding machine learning tools to monitor patient conditions persistently and predict side effects and overall outcomes. The current safety monitoring process described in the *Study Conduct* section relies on the EDC process. However, the Data and Safety Monitoring Board convenes only when the clinical trial has been conducted for a while and the data have met a certain point. Adding artificial intelligence components to the Study Conduct module can achieve more efficient monitoring. We will also investigate more potential in CTMS design using blockchain technology, such as integrating secure multiparty computation with blockchain for computational applications such as subject matching and using the cryptocurrency concept to build a novel CTMS that will help ensure timely validation and payment.

### Conclusions

In this study, we described a blockchain-based CTMS that covers 4 different stages of clinical trials. Through our simulation process, we empirically proved the feasibility of each application in the blockchain architecture. Compared with the scalability test on the Ethereum blockchain from our previous research, Quorum blockchain shows an overall better performance. The unique contribution of this work is the exploration of the benefits of blockchain technology in targeting the needs of CTMSs. This covers several essential functions (each of which is a part of the clinical trial process) using a distinctive blockchain adapter design to support an efficient, secure, traceable, transparent, and auditable management system. Our system design, implementation, and simulation results demonstrate the potential of blockchain to create a CTMS, and we suggest that this should serve as a notice for the health IT community to consider this emerging technology.

## Abbreviations

CRF: case report form
CTMS: clinical trial management system
eCRF: electronic case report form
EDC: electronic data capture
EHR: electronic health record
eTMF: electronic trial master file
GUI: graphical user interface
IPFS: interplanetary file system
SDV: source data verification
TMF: trial master file
TPS: transactions per second
